# 714 Safety and Efficacy of Enteral Nutrition During Burn Surgery: A Retrospective Chart Review

**DOI:** 10.1093/jbcr/irac012.268

**Published:** 2022-03-23

**Authors:** Danielle M Toepfer, Lacey Mangano, Niknam Eshraghi

**Affiliations:** Legacy Oregon Burn Center, Portland, Oregon; Legacy Oregon Burn Center, Portland, Oregon; Legacy Emanuel Medical Center, Portland, Oregon

## Abstract

**Introduction:**

Burn injuries are life-threatening conditions consisting of elevated oxidative stress, severe inflammation, and an increased hypermetabolic and catabolic state. It is recommended to start enteral nutrition (EN) early in the burn injured patients. These patients undergo many operations, and with stopping of EN the intermittent caloric deficit multiplies. This leads to increased risk of negative outcomes. We sought to analyze the efficacy of continuing EN feedings during burn surgeries to maximize caloric intake while also evaluating for adverse outcomes such as pneumonia.

**Methods:**

A retrospective chart review of intraoperative feeding and pneumonia rates in pediatric and adult burn patients admitted during 2011-2017 to a regional burn center was conducted. Inclusion criteria included patients with secured airway via endotracheal tube or tracheostomy, mechanical ventilation for at least four days, and those who received EN during operations. The 2017 National Burn Repository (NBR) was used as the control population with mechanical ventilation ≥4 days and a yes/no pneumonia diagnosis. Our assumption was that these patients likely did not receive intraoperative EN as this is not standard protocol at this time.

**Results:**

67 patients from our center and 779 NBR patients met inclusion criterion. EN were provided in 49-79% of total operations in the study group. The study group had a longer length of stay (LOS: 62 ± 34.4 vs 41 Days ± 31.7; p< 0.0001), Ventilator days (39 ± 28.9 vs 23 ± 43.9; p< 0.0001) and total body surface area burn percentage (TBSA: 36 ± 21 vs 31 ± 23 p-0.029).

The relationship between pneumonia, inhalation injury, COPD, diabetes, and current smoking status was examined in the case population. The odds of a patient with inhalation injury developing pneumonia was 2.83 times greater than a patient without an inhalation injury. There was a higher pneumonia rate in the study group compared to control population (56.7% vs 18.9%; p< 0.0001). However based on a multinomial logistics regression analysis, there was no statistically significant association between intraoperative feeding and pneumonia in the study group (p-0.597).

**Conclusions:**

We found a significant difference in the occurrence of pneumonia in our patient group compared to the NBR population, but our patients had higher LOS, ventilator days and TBSA burns. This suggest that our study group may have had higher acuity increasing risk of pneumonia. Our pneumonia rates do correspond with the rates reported from other comparable populations of burn patients. Despite this we did not find and association suggesting intraoperative feedings as the cause.

